# Fluvastatin-Pretreated Donor Cells Attenuated Murine aGVHD by Balancing Effector T Cell Distribution and Function under the Regulation of KLF2

**DOI:** 10.1155/2020/7619849

**Published:** 2020-12-19

**Authors:** Kai Zhao, Yu Tian, Junjie Wang, Chong Chen, Bin Pan, Zhiling Yan, Shengyun Zhu, Kailin Xu

**Affiliations:** ^1^Blood Diseases Institute, Xuzhou Medical University, Xuzhou, 221002 Jiangsu, China; ^2^Department of Hematology, The Affiliated Hospital of Xuzhou Medical University, Xuzhou, 221002 Jiangsu, China; ^3^Key Laboratory of Bone Marrow Stem Cell, Jiangsu Province, China; ^4^The People's Hospital of Jiaozuo City, Henan Province, China

## Abstract

Prevention of acute graft-versus-host disease (aGVHD) after allogeneic hematopoietic stem cell transplantation (allo-HSCT) is still to be explored. Statins are potent immunomodulatory agents that hold promise as novel and safe agents for aGVHD prophylaxis, yet the controversial effect and regulatory mechanism are incompletely understood. Here, in an MHC mismatched murine model, we found that Fluvastatin-pretreated donor cells could attenuate aGVHD severity by remission tissue pathological injury. Fluvastatin served to restrain effector T cells entry into aGVHD target organs from secondary lymphoid organs (SLOs). The potential mechanism of correcting the effector T cell biased distribution was that Fluvastatin elevated CD62L and CCR7 expression while decreased CXCR3 and CD44 levels, which were correlated with Kruppel-like factor 2 (KLF2) sustention in donor-derived cells. In addition, Fluvastatin was contributed to reducing cytokines IFN-*γ*, TNF-*α*, and granzyme-B production in allogeneic effector CD4^+^ and CD8^+^ T cells. Furthermore, evidence confirmed that Fluvastatin had a long-lasting effect to sustain KLF2 expression both in vitro and in vivo even under the stimulated circumstance. In conclusion, administration of Fluvastatin to donor mice showed protective effects against recipient aGVHD when compared to untreated mice due to the retention of effector T cells in lymphoid organs accompanying with reduction of nonlymphatic infiltration and related inflammatory cytokines.

## 1. Introduction

Inhibitors of 3-hydroxy-3-methylglutarylco enzyme A (HMG-CoA) reductase, also known as statins, have been proposed in allogeneic hematopoietic stem cell transplantation (HSCT) for their new promising roles on graft-versus-host disease (GVHD), infection after HSCT, and the efficacy of chemotherapy [1, 2]. Accumulating evidence showed that GVHD as the major complication of HSCT can be prevented or alleviated by various statin administrations both in animal and human studies [3–5]. Statins could affect the development of GVHD from many angels by inhibiting antigen presentation, development of Th1, and possibly Th17 cells and by enhancing the Th2 bias, different types of Treg cells, and stabilization of endothelial cells [6, 7]. However, based on the multiple effects of statins on the immune system, further mechanisms whereby statins may exert their effects on GVHD should be explored.

The pathological process of acute GVHD (aGVHD) is complex but involves the migration of T cells into secondary lymphoid organs (SLOs), and activated T cells migrate to aGVHD target organs, primarily the liver, gut, lung, and skin. Kruppel-like factor 2 (KLF2) is a member of a transcription factor family, which is proved essential for T cell trafficking by regulating T cell homing receptor CD62L, CCR7, and S1P1 for T cell recirculation through peripheral lymphoid organs [8]. Evidence showed that statin treatment of mouse and human T cells increased the expression of KLF2 through a HMG-CoA/prenylation-dependent pathway [9]. We therefore hypothesized that the immunomodulatory effects of statins on aGVHD are partly due to the regulation of T cell migration to recipients.

To determine of this, we treated mice by statins to test the changes of KLF2 levels in bone marrow (BM) and splenic cells. Then, aGVHD severity, effector T cell distribution, and function were evaluated in mice transplanted with Fluvastatin-pretreated donor-derived cells. In the present study, we show that statins upregulate KLF2 expression in a long-lasting pattern in vivo. Fluvastatin pretreatment on donor mice ameliorates aGVHD by decrease effector T cell infiltration into aGVHD target organs in MHC mismatched transplantation model.

## 2. Materials and Methods

### 2.1. Mice

Male C57BL/6 (H-2K^b^, 8~10 weeks old) and male BALB/c (H-2K^d^, 10~12 weeks old) mice were purchased from SLAC Laboratory Animal CO. Ltd, Shanghai in China. All animals were housed in a specific pathogen-free (SPF) facility. All the mice protocols were approved by the animal ethics standards.

### 2.2. BM Cell Treated by Statins In Vitro and KLF2 Detection

Fluvastatin (93957-55-2) and Simvastatin (79902-63-9) were purchased from Meilun Biotech Co. Ltd., Dalian, China, and dissolved into ddH_2_O and ethanol, respectively. C57BL/6 mice were sacrificed by cervical dislocation. BM cells from femurs and tibias were flushed out by a 23 G syringe needle, and T cells were depleted by Easy Sep CD90.2^+^ selection kit II (18951 stem cell). After cultured with Fluvastatin (200 *μ*mol/l) or Simvastatin (200 *μ*mol/l) for 18 h at 37 degree, BM cells were collected for detection of KLF2 expression. Other groups of BM cells stimulated by Fluvastatin or Simvastatin for 18 h and then activated by plate-bound anti-CD3 (4 *μ*g/ml, 16-0031, eBioscience) and soluble-CD28 (2 *μ*g/ml, 16-0281, eBioscience) for 6 h. Cells without anti-CD3 and CD28 stimulation were set simultaneously as a control. After treatment, BM cells were washed twice by phosphate-buffered saline (PBS), and KLF2 expression was assessed by Western blot.

### 2.3. Fluvastatin-Treated Mice In Vivo

C57BL/6 mice were injected intraperitoneally (20 mg/kg, 200 *μ*l/mouse) once daily for 7 consecutive days and then withdraw the Fluvastatin injection. Mice were sacrificed immediately on day 7, 3 days later after injection (10 d) or 10 days later (17 d). Bone marrow and spleen were harvested and stimulated by plate-bound anti-CD3 and soluble-CD28 for 6 h, respectively. Unstimulated controls were set simultaneously. Expressions of KLF2 on mRNA and protein levels were detected by RT-PCR and Western blot. There were 2 independent experiments (*n* = 15), and culture was done in triplicate.

### 2.4. Donor Mice Pretreated by Fluvastatin

C57BL/6 mice were used as donors, and the transplant day was set as day 0. Fluvastatin was injected intraperitoneally to C57BL/6 donors once daily from -7 days for consecutive 7 days before transplantation as above. The control donors were treated with an equal volume of solution buffer simultaneously. Fluvastatin-pretreated mice as donors were sacrificed to harvest BM and splenocytes for transplantation. Donor spleens were ground slightly on a 200 *μ*m mesh, and splenic lymphocytes were isolated by Ficoll (Dakewe, Shenzhen in China) for recipients. BM and SP were harvested, respectively, and the mRNA and protein levels of KLF2 were detected.

### 2.5. GVHD Model Establishment and Evaluation

GVHD models with different donor splenic cells were established according to our previous paper with mild revision [10]. Briefly, BALB/c recipients underwent total body irradiation (TBI) with 7.5 Gy of ^60^Co *γ* radiation. Recipients were cotransferred with BM cells (5 × 10^6^/mouse) and splenic cells (5 × 10^5^/mouse) from Fluvastatin-treated and control donors, which were named as GVHD+Fluvastatin group and GVHD+buffers group, respectively. An unmanipulated group referred to mice that had received neither TBI nor transplantation. The recipients were monitored daily for survival and every 3 days for body weight changes and clinical signs of aGVHD (hair loss, hunched back, activity, and diarrhea). To confirm the formation of chimera after transplantation, the bone marrow cells of recipients were detected every two days, and almost 90% cells were donor derived around day 14, which was consistent with our previous data [10]. Three independent experiments were repeated and 4-5 mice per time point from different groups were included.

### 2.6. Histopathology Scoring and Immunohistochemical Staining

Recipient mice were sacrificed on days 3, 7, 14, 21, and 28 after transplantation, and tissue samples (liver, lung, and gut) were fixed in 4% paraformaldehyde and subsequently embedded in paraffin. Paraffin-embedded tissue sections of 5 *μ*m thickness were stained by hematoxylin/eosin (H/E). Pathological damage and pathological score were evaluated according to the Cooke Histopathology Scoring System [11]. Besides, paraffin-embedded sections were used for immunohistochemical staining. Briefly, sections were deparaffinized, and antigens were retrieved using the microwave for 30 minutes. Slides were pretreated with 0.3% hydrogen peroxide (Beijing Zhongshan Golden Bridge Biotechnology Co., Ltd) for 10 minutes to neutralize endogenous peroxidase activity before blocked with goat serum for 30 min. Then, sections were incubated with unlabeled primary antibodies (CD3 mAb, 1 : 100, ab5690, Abcam, Cambridge, UK) overnight at 4°C. The sections were incubated with anti-rabbit HRP conjunct secondary Abs (Beijing Zhongshan Golden Bridge Biotechnology Co., Ltd.) for 1 hour at room temperature and DAB reagents (Beijing Zhongshan Golden Bridge Biotechnology, Co., Ltd.) were added subsequently. Photographs of stained tissue sections were obtained by microscope (Olympus BX-51, Japan), and CD3-positive cells were counted in each of the 4 fields from one section at 200-fold magnification.

### 2.7. Flow Cytometric Analysis (FACS) and Intracellular Cytokine Staining

Single-cell suspensions from the thymus, spleen, inguinal, and mesenteric lymph nodes and peripheral blood at different time points after allo-BMT were harvested. Surface markers were stained with conjugated mAbs including anti-mCD3-FITC (#555274), anti-mCD4-PerCP (#553052), and anti-mCD62L-APC (#561919), which were purchased from BD Pharmingen. Chemokine receptors mAbs anti-mCCR7-eFlour450 (#48-1971-82, eBioscience), anti-mCXCR3-PE-Cy7 (#25-1831-82, eBioscience), and anti-S1P1 (PE, # FAB7089P, R&D Systems) were used for T cell phenotype detection. For intracellular staining, splenic cells were incubated with or without PMA (50 ng/*μ*l, Sigma-Aldrich) plus ionomycin (1 *μ*g/*μ*l, Sigma-Aldrich) in the presence of Brefeldin A (10 mg/ml; Invitrogen) at 37°C for 4 h. Cells were washed and stained for CD3 and CD4 markers. Then, cells were fixed with 4% paraformaldehyde and permeabilized in Perm buffer (0.1% BSA, 0.05% NaN3, and 0.1% Saponin in PBS) overnight at 4°C. Intracellular cytokines IFN-*γ* (PE, #505808, Biolegend), TNF-*α* (APC, #17-7321-82, eBioscience), and Granzyme-B (eFluor450, #48-8898-82, eBioscience) were stained for 20 min in dark. Isotype controls and FMO were included in each staining. Cells were acquired from FACS Forteassa (BD Pharmingen, USA), and data were analyzed by Cellquest and Flowjo software.

### 2.8. Real-Time RT–PCR

Bone marrow cells and splenic mononuclear cells from individual mice were lysed, and mRNA was extracted. Real-time PCR was performed on LightCycler®480II (Roche), according to the manufacturer's instructions. The following primer sets were used: KLF2 F 5′-ACA GAC TGC TAT TTA TTG GAC CTT AG-3′, R 5′-CAG AAC TGG TGG CAG AGT CAT TT-3′; *β*-actin F 5′-TCC TTC GTT GCC GGT CCA-3′; R 5′-ACC AGC GCA GCG ATA TCG TC-3′. The level of KLF2 was determined relative to *β*-actin expression, which was used as an internal control in each sample.

### 2.9. Western Blot Analysis

BM cells and splenic mononuclear cells were collected after exposure to Fluvastatin or solution buffer in vitro or in vivo. Cells were lysed by Protein Extraction Reagent (Thermo), 1 mM with phenylmethanesulfonyl fluoride (PMSF), and 0.25% EDTA and then incubated on ice for 20 min. Lysates were centrifuged at 20000 g for 15 min at 4°C. Supernatants were mixed with one-quarter volume of 4x SDS sample buffer, boiled for 10 min, and then separated by sodium dodecyl sulfonate-polyacrylamide gel electrophoresis (SDS-PAGE) in Tris-HCl Ready-Gels. Proteins were transferred to a nitrocellulose filter membrane (GE Healthcare, Whatman), and immunoblotting was performed. Primary protein antibodies KLF2 (Abcam company) and GAPDH (Bioworld Technology) were used in blocking buffer overnight at 4°C. The membrane was incubated with goat anti-rabbit IgG antibody (Cell Signaling Technology) for 1 h at RT. After extensive washing, proteins were visualized by an ECL-Plus Kit, and the blots were exposed to a Kodak radiographic film.

### 2.10. Cytometric Bead Array Analysis

Plasma from each of the groups was obtained for 3, 7, 14, 21, and 28 days following transplantation, respectively. Cytokine production of IFN-*γ*, TNF-*α*, IL-4, and IL-10 and chemokines MCP1, MCP3, MIP-1*α*, MIP-1*β*, IP-10, CXCL9, and RANTES were measured with a cytometric bead array (CBA) using flow cytometry, according to the manufacturer's instructions.

### 2.11. Statistical Analysis

Data were expressed as mean values ± standard deviation (SD). Statistical analyses were performed using the 2-tailed Student's *t* test for two-group comparisons. ANOVA with Tukey's Multiple Comparison test was used for three-group experiments. *P* values ≤ 0.05 were considered statistically significant.

## 3. Results

### 3.1. KLF2 Expression Can Be Upregulated by Statins in a Long-Term Way

**Figure 1:**
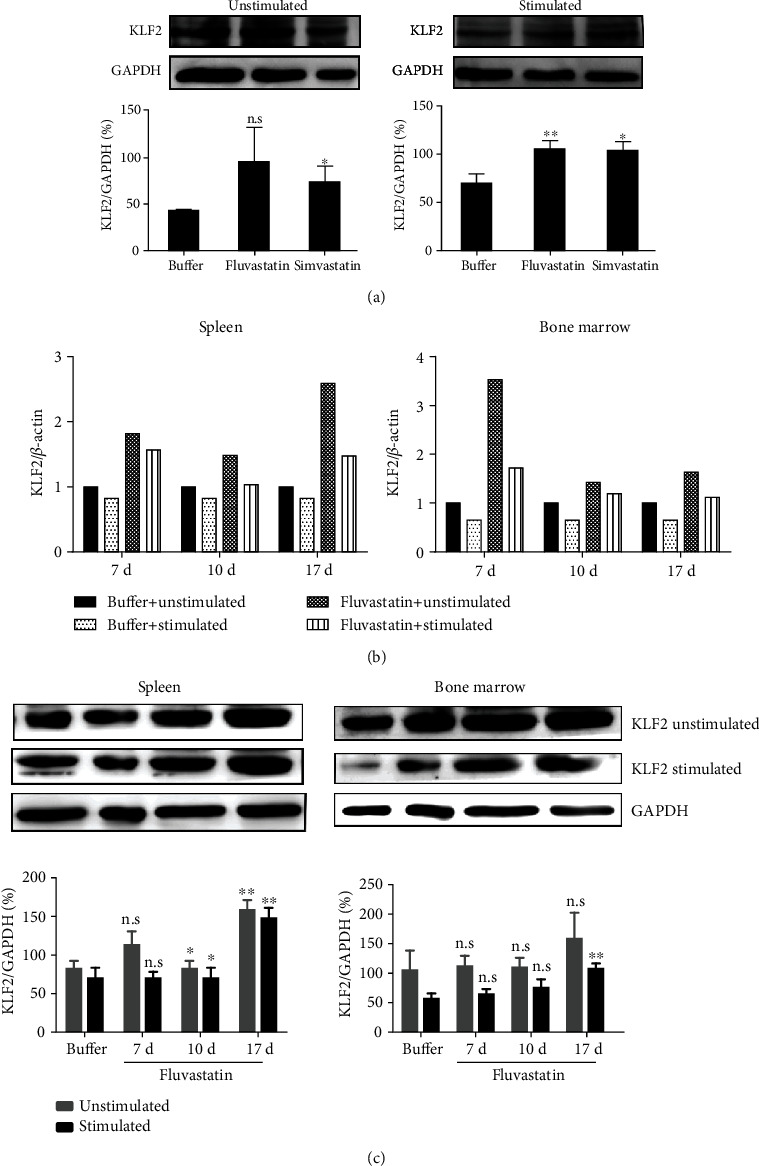
The expression of KLF2 was increased after statin treatment in vitro and in vivo. (a) Bone marrow cells of C57BL/6 mice were cultured with Fluvastatin (200 *μ*mol/l) or Simvastatin (200 *μ*mol/l) or equal volume of PBS for 18 hours and stimulated with or without plate-bound anti-CD3 plus soluble-CD28 in vitro for additional 6 hours. Then, cells were collected for detection of KLF2 expression by Western blot. Experiments were repeated twice, and the culture was set up in triplicate. Cells in statin-treated groups were compared to those in buffer controls, and statistical analysis was done. (b) C57BL/6 mice were daily i.p. injeced of Fluvastatin or PBS (*n* = 15) for 7 consecutive days and withdraw drug administration, and then, on days 7, 10, and 17, mice were killed to collect samples. KLF2 mRNA and protein levels (c) in bone marrow cells and spleen cells which were stimulated with or without anti-CD3 and anti-CD28 were analyzed by qRT-PCR and Western blot, respectively. Two independent experiments were repeated. Cells collected at each time point in Fluvastatin groups were compared to those in the buffer controls. ^∗^*P* < 0.05, ^∗∗^*P* < 0.01, n.s *P* > 0.05.

To examine the effects of statins on KLF2 expression, BM cells separated from C57BL/6 mice were treated with or without statins in vitro. As shown in Figure 1(a), both Fluvastatin and Simvastatin treatment of BM cells increased KLF2 expression. Furthermore, higher levels of KLF2 were still maintained at the presence of statins in culture 6 hours after initiation of anti-CD3 and anti-CD28 stimulation (Figure 1(a)). Then, we ask if statins can play a long-lasting effect on KLF2 expression in vivo. C57BL/6 mice were daily intraperitoneal injected with Fluvastatin or buffer for 7 consecutive days and then statin withdrawal. Spleen and BM were harvested and analyzed on day 7, day 10, and day 17, respectively. As we expected, Fluvastatin blocked downregulation of *Klf2* mRNA in naïve and activated splenetic cells on day 7. Even though there were not Fluvastatin administration on day 10 and day 17, *Klf2* mRNA was still sustained at higher levels compared to the control group (Figure 1(b)). Additionally, we found that Fluvastatin significantly elevated *Klf2* mRNA in BM cells at low levels in buffer-treated mice on day 7. In the absence of Fluvastatin, *Klf2* mRNA was continued to keep higher levels on day 10 and day 17. Following the increased *Klf2* mRNA, the elevated protein levels of KLF2 were shown on day 10, and this long-term influence persisted until at least day 17. The similar phenomenon was investigated for KLF2 protein expression in BM cells especially in stimulated cells (Figure 1(c)). Taken together, our results indicated that statin treatment could upregulate the expression of KLF2 and further prevent KLF2 degradation after activation. In addition, Fluvastatin had a long-lasting effect on KLF2 expression, which was independent on continuous statin exposure.

### 3.2. Donor Pretreatment with Fluvastatin Plays Protective Roles in aGVHD Progress

**Figure 2:**
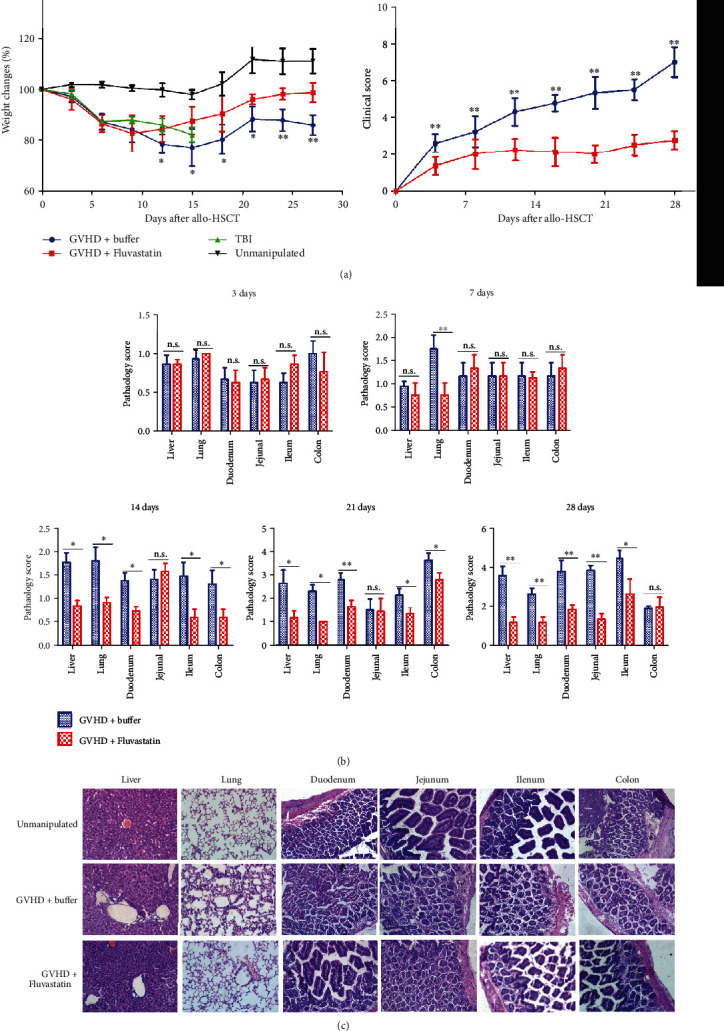
Transferring with Fluvastatin-pretreated donor cells improved the general conditions and alleviated tissue pathological injury in recipient after transplantation. (a) Body weight changes were quantified and clinical score including hair loss, posture, mobility, and diarrhea were assessed in recipients at series of time points posttransplantation. (b) Recipients were monitored and were sacrificed on different days. Pathological score of recipient target organs (liver, lung, and guts) were measured by HE stating and (c) HE photographs (×200) of recipient target tissue slices from each group on 28 days after transplantation were shown. Data represents the mean ± SD of results from 3 independent experiments. Mice were included for serial time point detections in GVHD with or without Fluvastatin-pretreated donor cells in every repeated experiment (*n* = 21), respectively. TBI or unmanipulated groups were used as control (*n* = 4). ^∗^*P* < 0.05, ^∗∗^*P* < 0.01, n.s *P* > 0.05.

Based on the data of KLF2 upregulation in vivo by statins, we predicted that Fluvastatin would alleviate aGVHD severity of recipients transplanted with pretreated donor-derived cells. C57BL/6 mice as donors were daily ip injected with Fluvastatin for 7 days, and then, the BALB/c recipients were transplanted with pretreated or unpretreated donor BM and splenic cells. As shown in Figure 2(a), recipients with Fluvastatin-pretreated donor cells named as GVHD+Fluvastatin group showed gradual weight recovery from day 9 and displayed significantly less weight loss compared with transplanted mice without pretreated donor cells (GVHD+buffer group) since day 12 (days 12, 15, 18, and 21 showed *P* < 0.05 and days 24 and 27 showed *P* < 0.01). According to the aGVHD clinical signs including hair loss, hunched back, diarrhea, and reduced mobility, obvious lower clinical scores were recorded for GVHD+Fluvastatin recipients than that for GVHD+buffer group (Figure 2(a)). To further learn about the impacts of donor statin pretreatment on the pathological changes of aGVHD target organs, tissue slices were stained with HE, and the pathological score was evaluated. The results in Figure 1(b) showed that no obvious difference was investigated on day 3 between the two groups, while a mild high score from GVHD+buffer mice was observed only in the lung on day 7 (*P* < 0.001). The pathological score of GVHD+Fluvastatin mice kept at lower levels, while almost all of GVHD+buffer mice target organs (liver, lung, small intestines, and colon) showed increased score on day 14, day 21, and day 28 (*P* < 0.05 and *P* < 0.001). Slices on day 28 in Figure 1(c) represented that more severe damage of the liver, lungs, and gut was observed in mice of GVHD+buffer group compared with that in GVHD+Fluvastatin group. Hepatic central veins in GVHD+buffer mice were remarkably surrounded by inflammatory cells. Vascular inflammation, perivascular and peribronchiolar cuffing, and alveolar hemorrhage were clearly shown in the lung from GVHD+buffer mice, while slight lesion and normal alveolar were shown in GVHD+Fluvastatin group. The higher number of mononuclear and granulocytic cells infiltrated into the lamina propria in control mice than that in the group with Fluvastatin pretreatment. In conclusion, compared with GVHD+buffer mice, significant protection from serious GVHD was investigated in mice receiving Fluvastatin-pretreated donor cells. The data confirmed that donor pretreatment in vivo with Fluvastatin conferred aGVHD protection.

### 3.3. The Distribution of T Cells in Allo-HSCT Recipients Receiving Fluvastatin-Pretreated Donor Cells

**Figure 3:**
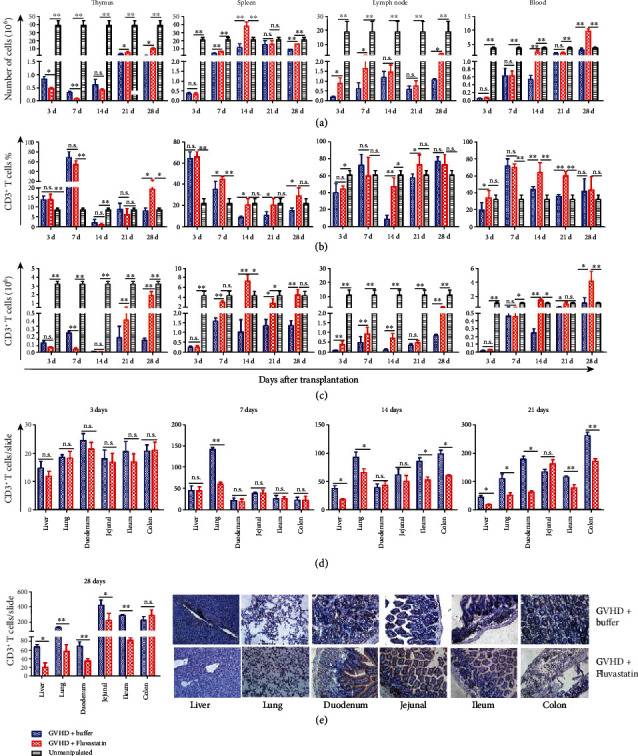
Fluvastatin pretreatment adjust migration ability of donor derived cells. (a) Total mononuclear cell number. (b) Flow cytometric analysis of CD3^+^ T cell proportion and (c) absolute number of CD3^+^ T cells in thymus, spleen, lymph nodes, and blood from recipients receiving Fluvastatin-pretreated or Fluvastatin-unpretreated donor cells. (d) Infiltration of CD3^+^ T cells in GVHD target organs were measured (cell number/section) by immunohistochemical staining on days 3, 7, 14, 21, and 28 (e) posttransplantation. Slices of target organs were shown (×200). Data represents the mean ± SD from 3 independent experiments (*n* = 4 − 5 per time point). ^∗^*P* < 0.05, ^∗∗^*P* < 0.01, n.s *P* > 0.05.

We next investigated the mechanisms by which Fluvastatin alleviates aGVHD. The total mononuclear cells in lymphatic organs and blood of recipients with Fluvastatin-pretreated or Fluvastatin-unpretreated donor cells were recovered on days 3, 7, 14, 21, and day 28 after transplantation. Total thymic cell numbers were lower in GVHD+Fluvastatin group on day 3 and day 7 than those in GVHD+buffer group; however, the former increased faster since day 21 (Figure 3(a)). Much more cells gathered in the spleen and lymph nodes of GVHD+Fluvastatin mice compared to recipients receiving buffer-pretreated donor cells during aGVHD progression (Figure 3(a)). Peripheral circulation of GVHD+Fluvastatin also contained higher cell numbers on day 14 and day 28.

Flow cytometry analysis showed that thymic CD3^+^ T cell proportion elevated higher in GVHD+Fluvastatin mice than that in GVHD+buffer mice until day 28, while absolute CD3^+^ T cell number significantly increased since day 21 (Figures 3(b) and 3(c)). Fluvastatin pretreatment also promoted the percentages and absolute numbers of CD3^+^ T cells in the spleen. Higher CD3^+^ T cells were kept in lymph nodes and blood in GVHD+Fluvastatin group when compared with that in GVHD+buffer group at serial time points (Figures 3(b) and 3(c)). Therefore, next, we aimed to investigate the distribution of CD3^+^ T cells in nonlymphoid organs. GVHD target organs were harvested to make paraffin slices, and IHC was used to identify infiltrated CD3^+^ T cells. Figure 3(d) showed that there was no difference between GVHD+Fluvastatin and GVHD+buffer groups 3 days after transplantation. However, on day 7, obvious higher numbers of infiltrated CD3^+^ T cells in GVHD+buffer mice were found in the lung, which was consistent with the early pathological damage in the lung shown in Figure 2(b). As GVHD continued to advance, much more CD3^+^ T cells migrated into the peripheral target tissues of control mice, but recipients transferred with Fluvastatin-pretreated donor cells had fewer CD3^+^ T cell infiltration in the liver, lung, small intestine, and colon (Figures 3(d) and 3(e)). These results suggest that Fluvastatin pretreatment of donor cells was important for the accumulation of allo-reactive T cells in the lymphatic circulatory system and prevented their pathological infiltration into aGVHD target organs.

### 3.4. The Expression of Receptors Related to the Migration of Fluvastatin-Treated T Cells

**Figure 4:**
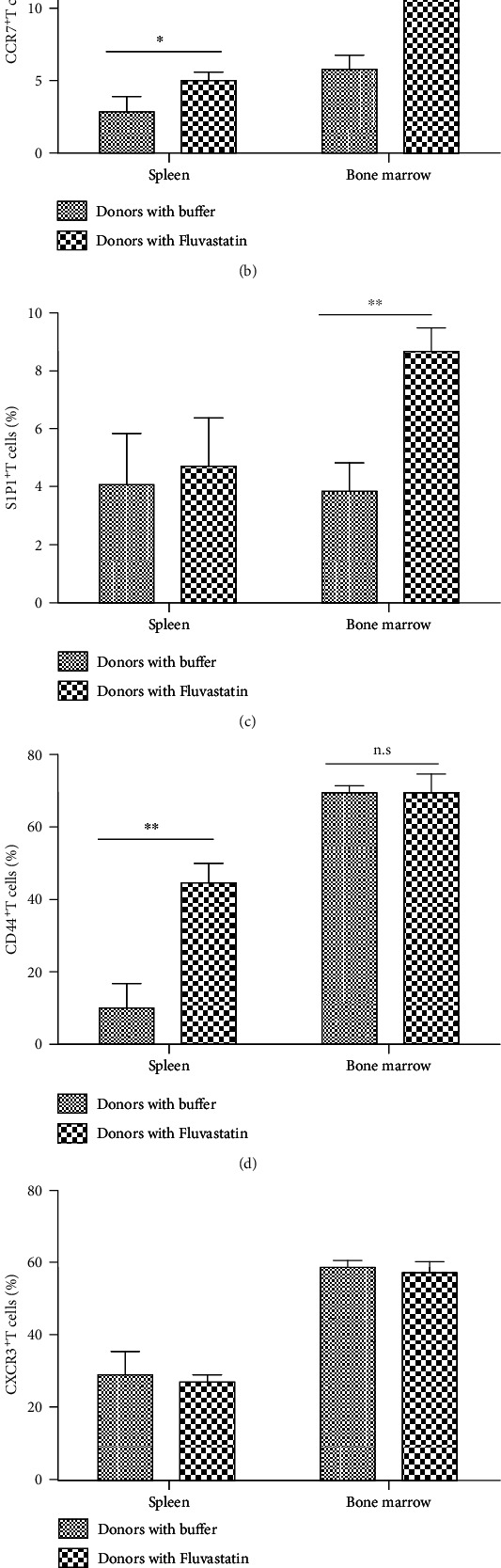
The expression of molecules related to T cell migration after Fluvastatin treatment in vivo. C57BL/6 mice were intraperitoneally injected with Fluvastatin or an equal volume of buffer daily for 7 consecutive days before sacrificed. The expression of (a) CD62L, (b) CCR7, (c) S1P1, (d) CD44, and (e) CXCR3 on spleen and bone marrow T cells were detected by flow cytometry. (f) The proportion of CD3^+^ T cells in the spleen and bone marrow from buffer- and Fluvastatin-treated mice were explored by flow cytometry. Data represents the mean ± SD of results from 3 experiments (*n* = 5 per group). ^∗^*P* < 0.05, ^∗∗^*P* < 0.01, n.s *P* > 0.05.

To further understand the mechanism of altered trafficking patterns in Fluvastatin-pretreated cells, surface markers related to T cell migration were detected. C57BL/6 mice were ip injected Fluvastatin or buffer for consecutive 7 days, and then, spleen and bone marrow were harvested. The results in Figure 4 showed that CD62L and CCR7 as homing receptors for T cell entry into SLOs were both upregulated in mice treated with Fluvastatin. S1P1-mediated egress of T cells from SLOs showed no difference in spleen while higher expression was detected in the BM of Fluvastatin-injected group. Increased adhesion molecule CD44-positive T cells were shown only in the spleen, while there was no statistically significant difference in CXCR3 expression neither in the spleen nor in the BM (Figures 4(d) and 4(e)). The above data suggested that Fluvastatin in vivo treatment induced T cells gained the potential travelling ability to SLOs but had no influence on chemokine receptor expression. Simultaneously, the proportions of CD3^+^ T cells in the spleen and BM from buffer- and Fluvastatin-treated mice were found at similar levels, which identified that the percentage differences of above surface receptor positive T cells were not due to the change of total CD3^+^ T cells (Figure 4(f)).

### 3.5. The Kinetics of Trafficking-Related Surface Molecules on Effector T Cell in Recipients Receiving Fluvastatin-Pretreated Donor Cells

**Figure 5:**
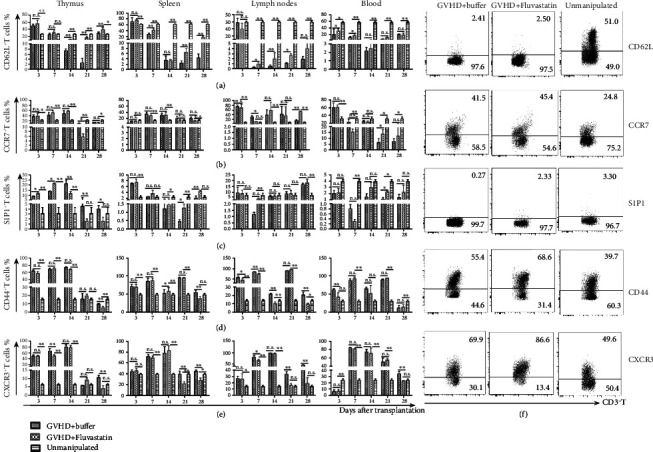
The expression of surface molecules related to T cell migration on T cells in transplanted recipients. BALB/c mice were transplanted with bone marrow plus T cells from Fluvastatin or buffer pretreated C57BL/6 donors. Single-cell suspension of the thymus, spleen, lymph nodes, and blood were acquired from each recipient on days 3, 7, 14, 21, and day 28 posttransplantation and subsequently gated on CD3^+^ T cells by flow cytometry to analyze (a) CD62L, (b) CCR7, (c) S1P1, (d) CD44, and (e) CXCR3 expression. Data represents the mean ± SD of results from 3 independent experiments. There were 4-5 mice in each time point in Fluvastatin and buffer GVHD groups, respectively, and 4 mice were sacrificed in unmanipulated group. ^∗^*P* < 0.05, ^∗∗^*P* < 0.01, n.s *P* > 0.05. (f) One representative FACS data of the spleen on day 14 from 3 independent experiments was shown, and the number in the corner means the population percentage.

To determine the variation of migrating molecules from effector T cells in recipients following Fluvastatin-pretreated donor cells transplantation, recipient mice with aGVHD were sacrificed at serial time points. Results showed in Figure 5(a), compared to aGVHD mice receiving untreated donor cells, GVHD+Fluvastatin mice possessed a higher percentage of CD62L^+^ CD3^+^ T cells in the early stage in blood and in SLOs (day 3 and day 7). Thymic CD62L^+^ CD3^+^ T cells increased since day 14, which might be due to the regeneration of transplanted Fluvastatin-pretreated bone marrow cells. Homing receptor CCR7 was increased on day 21 in blood and on day 28 in lymph nodes, while no significant difference in the other organs (Figure 5(b)). Expression of S1P1 was elevated on thymic T cells in GVHD+Fluvastatin group on days 3 and 7, whereas they were reversed on days 14, 21, and 28 posttransplantation. Though T cells in the spleen and blood from GVHD+Fluvastatin group showed a higher expression of S1P1 on day 21, while S1P1^+^ T cells localized in lymph nodes were found no distinction (Figure 5(c)). Evidence has showed that T cell activation in SLOs induced downregulation of S1P1 and S1P1 modulated cyclically in cell surface levels as lymphocytes recirculate from the blood into SLOs and then into blood or lymph [12]. Taken together, the above data suggested that the impress of Fluvastatin pretreatment on donor cells can be adoptively transferred to recipients, and furthermore, it can last a long time in vivo. CD44^+^ effector T cells were showed lower levels in blood on day 3 and kept lower degrees in thymus and SLOs on day28 in GVHD+Fluvastatin mice (Figure 5(d)). CXCR3, as the key chemokine receptor inducing allo-reactive effector T cells trafficking to multiple aGVHD target organs, was prevented on T cells from recipients receiving Fluvastatin-pretreated donor cells. Especially at the most serious stage of GVHD from day 21 to day 28, significantly fewer CXCR3^+^ T cells were found in SLOs of GVHD+Fluvastatin mice compared to control mice (Figure 5(e)). Representative flow plots data in Figure 5(f) showed the T subpopulations. These data may partly interpret the reason of retention of effector T cells in the lymphatic circulatory system and reduced infiltration to aGVHD target organs in recipients following Fluvastatin-pretreated donor cell transplantation.

### 3.6. Decreased Secretion of Inflammatory Cytokines in Effector T Cells from Recipients after Fluvastatin Pretreated-Donor Cell Transplant

**Figure 6:**
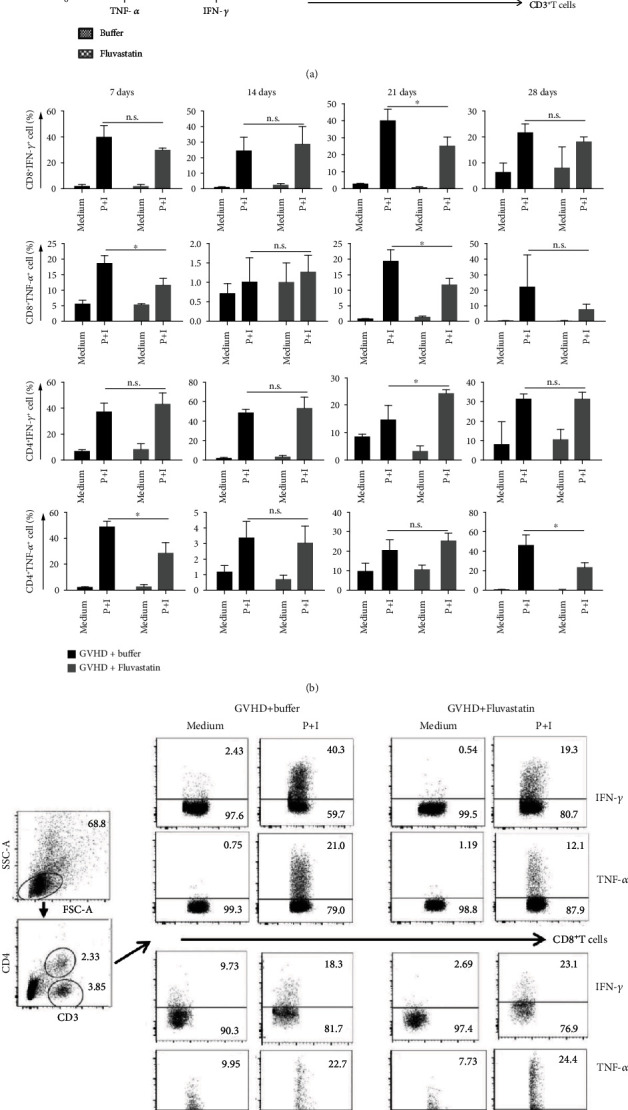
Cytokines production after Fluvastatin treatment. (a) Splenic mononuclear cells from C57BL/6 mice treated with Fluvastatin or buffer for consecutive 7 days were harvested. Cells were cultured with PMA (50 ng/*μ*l), ionomycin (1 *μ*g/*μ*l) for 3-5 hours, and brefeldin A (5 *μ*g/*μ*l) was added before flow cytometric analysis of TNF-*α* and IFN-*γ* production in CD3^+^ T cells. One representative FACS data from 3 independent experiments was shown in the right panel, and the number in the corner represents the percentages of gated cells. (b) BALB/c mice were used as recipients following transplantation of Fluvastatin- or buffer-pretreated C57BL/6-derived bone marrow and splenic cells. Splenic cells from recipients were stimulated as described above, and then, the production of TNF-*α* and IFN-*γ* in CD8^+^ and CD4^+^ T cells on days 7, 14, 21, and 28 were detected. Data represents the mean ± SD of results from 3 experiments (*n* = 4 − 5 per time point). ^∗^*P* < 0.05, ^∗∗^*P* < 0.01, n.s *P* > 0.05. (c) Representative FACS plots on day 21 were shown. The number in the corner means the cell percentages.

We next explored the influence of Fluvastatin on the functional responses of T cells. Two mice models were used here. First, we injected Fluvastatin to C57BL/6 mice for consecutive 7 days as we described above, and then, spleens were harvested for flow cytometry detection. We found that IFN-*γ* production was significantly inhibited in splenic T cells derived from Fluvastatin injected mice than that in buffer alone treated mice, while TNF-*α* expression was not changed (Figure 6(a)). These data suggested that effect or memory T cell responses could be suppressed by Fluvastatin. Then, BALB/c mice transplanted with BM and T cells from Fluvastatin- or buffer-pretreated donor cells were detected. Statistically decreased levels of IFN-*γ* and TNF-*α* in spleen CD8^+^ T cells from GVHD+Fluvastatin as compared with that in GVHD+buffer group were observed on day 21 after transplantation (Figures 6(b) and 6(c)). Though mild higher IFN-*γ* was shown in CD4^+^ T cells on day 21, lower TNF-*α* was detected on day 7 and day 28 in Fluvastatin-treated mice (Figure 6(b)). The attenuated ability of cytokine expression may be another reason for the alleviated GVHD severity in mice receiving Fluvastatin-pretreated donor cells.

### 3.7. Dynamics of Cytokines and Chemokines in Serum in Recipients

**Figure 7:**
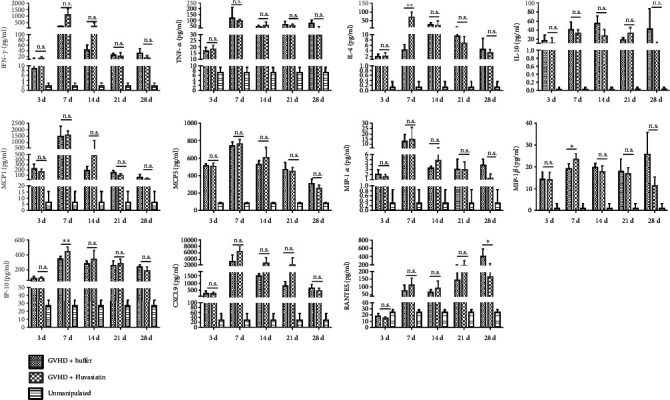
Systemic levels of cytokine and chemokine after Fluvastatin-pretreated donor cell transplantation. Recipients with Fluvastatin-pretreated and Fluvastatin-nontreated donor cells were killed for serum collection at different time points. CBA was used to detect the levels of cytokines and chemokines induced in GVHD mice, and unmanipulated mice serum was included as control. Each time point had 4-5 transplanted mice in 3 repeated experiments. ^∗^*P* < 0.05, ^∗∗^*P* < 0.01, n.s *P* > 0.05.

Furthermore, we explored the changes of systemic cytokine and chemokine levels in peripheral blood in recipients after Fluvastatin-pretreated donor cell transplantation. As shown in Figure 7, serum levels of IFN-*γ* and TNF-*α* in serum showed no difference between Fluvastatin-pretreated or Fluvastatin-nonpretreated transplanted mice. However, significantly increased IL-4, known as anti-inflammatory cytokine, was detected in GVHD+Fluvastatin group on day 7. Chemokine MCP1 and MCP3 as the monocyte attractor obviously increased after transplantation, but there is no difference between recipients with or without Fluvastatin pretreatment. MIP-1*β* as a ligand of CCR5 elevated on day 7 in GVHD+Fluvastatin mice, while MIP-1*α* showed no significant difference. Meanwhile, a higher level of IP-10 as the IFN-*γ* downstream proteins was investigated on day 7, which can recruit CXCR3^+^ T cells. CXCL9, another ligand of CXCR3, showed an increased trend in aGVHD mice, but no significant statistical difference was found between GVHD+Fluvastatin and buffer control. RANTES (also named CCL5), playing an important role in homing and migration of effector and memory T cells, showed a lower level in recipients with Fluvastatin-pretreated donor cells on day 28. The above data demonstrated that Fluvastatin pretreatment on donor cells also can regulate the expression of anti-inflammatory cytokines and some chemokines. The results suggested that higher chemokine levels in serum provided opportunities for effector T cells enrichment in the peripheral blood circulation system, which was also consistent with reduced tissue infiltrated T cells in our previous experiments.

## 4. Discussion

Although statins have been used from bench to bedside in allo-HSCT patients, many potential mechanisms were still unclear [1, 13]. Results from basic research studies will help to answer some of these questions and guide reasonably clinical therapy in the preparation process of donors and patients following allo-HSCT. Several studies on the correlations between statin use and risk of GVHD were conducted, and a protective effect of statin on GVHD was demonstrated [6]. Zeiser et al. reported in a mouse MHC-mismatched hematopoietic cell transplantation (HCT) model that dealt either donors or recipients with atorvastatin for 10 days before transplantation prevented recipient mice from aGVHD lethality by Th2 polarization and inhibition of uncontrolled Th1 response while maintaining GVL activity [3]. Donor rather than recipient's statin treatment protected against grades 3-4 aGVHD was observed in a retrospective analysis among 567 patients who had HCT from human leukocyte antigen-identical sibling donors [13]. These results indicated that donor statin use may be a feasible, safe, and potentially effective strategy to prevent aGVHD. However, multiple possible mechanisms that need to be defined were involved in statin-mediated aGVHD remission. Wang et al. found that lovastatin prevented GVHD by virtue of interfering with LFA1-mediated homing of T cells to GVHD target organs [14]. Here, we detected the role of statin on donor cells to the distribution of effector T cells during GVHD process, and the potential mechanism-drove effector T cell migration were explored.

Previous evidence indicated that statins could increase KLF2 expression in T cells and prevent activation-induced KLF2 reduction through the prenylation-dependent pathway [9]. Furthermore, KLF2 plays a pleiotropic effect on T cell migration by regulating the expression of certain surface receptors on T cells [8, 15]. Thus, we had a hypothesis that the potential mechanism of statin preventing aGVHD by regulating T cell distribution might be correlated with the level of KLF2 in effector T cells.

Here, our studies in vitro and in vivo confirmed that KLF2 expression in donor T cells was elevated, and the reduction of KLF2 expression after T cell activation was blocked after Fluvastatin treatment. Gene expression profiles showed that KLF2 gene level was downregulated, which could be contributing to the aggravation of immune injury in aGVHD, but there is no further study on their relationship [16]. Although the expression was reduced in mice of aGVHD, a noteworthy enhancive expression of KLF2 in spleen T cells derived from donors pretreated with Fluvastatin rather than buffer treatment was observed, and this adoptive transferred upregulated pattern was independent on the consecutive usage of statins to recipients.

In the present study, mice transferred Fluvastatin-pretreated donor cells possessed low-grade aGVHD, including lower clinical score and pathological score as well as milder host tissue lesions related to aGVHD with fewer inflammatory cells infiltrated in target organs after transplantation. Moreover, our study on the distribution of T cells in GVHD+Fluvastatin recipients also showed a decreased accumulation of allogeneic CD3^+^ T cells in the liver, lung, and intestine while more donor-derived CD3^+^ T cells migrated to the thymus, spleen, and lymph node and circulated among blood compared with recipients in GVHD+buffer group. The above data showed the role of statin on the regulation of effector T cell distribution following allo-HSCT, which was a novel perspective on how statin works in aGVHD.

We further determined the possible mechanisms of statins involved in aGVHD protection by analyzing the expression of adhesion molecules and chemokine receptors which were responsible for T cell trafficking. The previous reports demonstrated that KLF2-deficient T cells lead to reduced levels of CD62L and S1P1 and the upregulation of CXCR3 [17, 18]. The latest study showed that KLF2-sufficient Tregs treated with simvastatin decreased the expression of receptors associated with tertiary tissues (CCR4, CCR8, CCR9, and CD44) while simultaneously increasing the expression of molecules (CCR7, CD62L, and S1P1) used to circulate throughout SLOs [19]. However, there was no evidence showed in allo-HSCT recipients whether statin could induce T cell redistribution between lymphoid organs and aGVHD target organs. As expected, increased levels of CD62L and CCR7 were observed in T cells from Fluvastatin-treated donors as well as in GVHD+Fluvastatin recipients. The homing receptors CD62L and CCR7 facilitate T cells entry into lymphoid tissues, while S1P1 is required for mature T cells egressing from thymus and peripheral lymphoid organs, all of which attribute to keep the balance of the immune system [20–23]. Donor T cells derived from CD62L gene-knockout mice failed to migrate to peripheral lymph nodes to recipients, thus could not induce aGVHD after transplantation [24]. Lack of CCR7 on T cells induced less SLOs entry can attenuate aGVHD severity [25, 26]. Given that, Fluvastatin-induced increase of T cell homing might be potentially unfavorable in aGVHD, which seemed to be contradicting with the observation of reduced injury-related aGVHD in our report. However, some data suggested a more complicated set of functions of CD62L and CCR7 in aGVHD. Anderson argued against this concept in his investigation showed that T cell deficient in CD62L or CCR7 and transplanted recipients lacking PNAd ligands for CD62L, or recipients lacking all major secondary lymphoid tissues could induce aGVHD all the same [27]. Furthermore, our data showed lower S1P1 in thymus, and a similar level in draining LN indicated even though higher CD62L and CCR7 T cells were driven into SLOs, less effector T cells went out from SLOs which was in line with the previous evidence [28]. In addition, Hashimoto et al. provided other potential mechanism that retention of donor-derived T cells in LN by FTY20 increased the activation-induced apoptosis due to enhanced T cell/APC interaction contributing to GVHD amelioration [29]. More importantly, in our report, Fluvastatin pretreatment was correlated with lower expression of the activated molecule CD44 and inflammatory chemokine receptor CXCR3 on T cells in the recipient, which indicated a state of less activated and weakened ability of induce effector T cells to infiltrate into target organs, hence resulting in the milder tissue lesion in the host. The above data powerfully suggested that statins might change the levels of homing receptors and inflammatory chemokines by altering KLF2 expression, thereby shifting the balance of allo-reactive T cells between inflammatory and homeostatic migration in aGVHD.

In conclusion, in this paper, we presented evidence that KLF2 expression in donor T cells was upregulated after Fluvastatin treatment, and recipients transferred with donor cells of high expression of KLF2 after Fluvastatin pretreatment were developed milder tissue pathological injury associated with aGVHD. The potential mechanisms of KLF2-induced protective effects in aGVHD in our reports including beneficial distribution of donor-derived T cells in recipients with increased expression of CD62L and CCR7, while CXCR3 and CD44 expression was decreased on these T cells, which was responsible for the promotion of T cell homing and circulation as well as inhibiting migration of donor-derived T cells to host target organs. Moreover, allogeneic activation of donor-derived T cells and production of proinflammatory media such as TNF-*α* and (or) IFN-*γ* by donors and recipients were partly inhibited by statins, which also played an important role in aGVHD prevention.

## Data Availability

The data used to support the findings of this study are included within the article.
